# Positive and negative behavioural intentions towards refugees in Turkey: The roles of national identification, threat, and humanitarian concern

**DOI:** 10.1002/casp.2354

**Published:** 2018-06-21

**Authors:** Şenay Yitmen, Maykel Verkuyten

**Affiliations:** ^1^ Ercomer, Social and Behavioral Sciences Utrecht University Utrecht Netherlands

**Keywords:** behavioural intentions, humanitarian concern, national identification, perception of threat, refugees

## Abstract

The present research investigated positive and negative behavioural intentions towards Syrian refugees in Turkey. The behavioural intentions were examined in relation to national identification, perception of threat, and humanitarian concerns. A questionnaire was conducted among Turkish participants (*n* = 605) and the results showed that respondents made a distinction between negative and positive behavioural intentions towards Syrian refugees. Further, higher national identification was associated with more negative and less positive behavioural intentions, and perception of threat was responsible for these associations. In addition, humanitarian concern was associated with more positive behavioural intentions and less negative ones. Additionally, stronger humanitarian concern weakened the association between threat perceptions and negative behavioural intentions but also strengthened the association between higher threat and lower positive behavioural intentions.

## 1 INTRODUCTION

The past decade has seen a dramatic increase in the number of people who are forced to flee their home because of conflicts and wars (Edwards, 2015). The resulting adaptation challenges for both host societies and refugees have become a crucial issue that receives increasing attention in the social and behavioural sciences, including social psychology (see Esses, Hamilton, & Gaucher, 2017). Research has reported that some members of the hosting societies perceive refugees as a symbolic, security, and economic threat and as a result have negative attitudes towards refugees (Goot & Watson, 2005; Louis, Duck, Terry, Schuller, & Lalonde, 2007; Schweitzer, Perkoulidis, Krome, Ludlow, & Ryan, 2005). Yet other host society members have humanitarian concerns that makes them care about the fate of the innocent victims of conflict and disaster (e.g., Nickerson & Louis, 2008).

**Figure 1:**
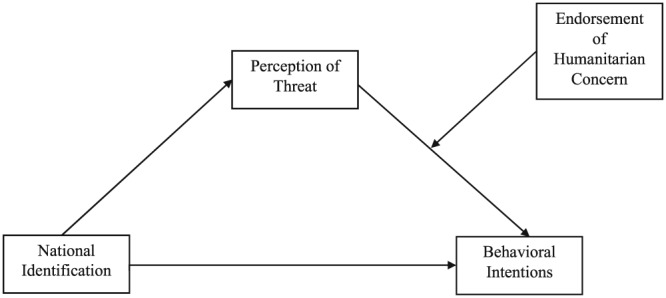
Moderated mediation model being tested

This study, conducted in Turkey, examines among self‐identified Turkish citizens positive and negative behavioural intentions towards Syrian refugees and whether these intentions are associated with national identification, perception of threat, and humanitarian concerns. It is investigated whether stronger humanitarian concern is associated with more positive behavioural intentions and less negative behavioural intentions. Additionally, we examined whether perception of threat mediates the relationship between national identification and positive and negative behavioural intentions and whether the role of threat depends on humanitarian concern. This allows us to find out whether national identification is related to positive and negative behavioural intentions because of perceptions of threat and whether this is conditional upon humanitarian concern (moderated mediation model; see Figure 1). The test of the conditional effect is based on the reasoning that feelings of threat will be less likely to be associated with (negative) behavioural intentions when there are at the same time humanitarian concerns about the welfare of Syrian refugees.

### 1.1 Refugees in Turkey

The conflict in Syria escalated rapidly in 2012 especially when efforts to negotiate a ceasefire failed (İçduygu, 2015). As a result, the number of Syrian refugees increased dramatically from 8.000 to 2.7 million in 5 years (UNHCR, 2016). Although legally, the Syrians were not refugees and did not benefit from refugee rights because of Turkey's geographical limitation to the *1951 Convention Relating to the Status of Refugees*, Turkey granted temporary protection status that included three main principles: an open‐door policy to Syrian refugees, no forced return to Syria, and unlimited length of stay in Turkey (Kaya, 2016). Turkey settled refugees in camps neighbouring the Turkish‐Syrian border (Kaya & Kıraç, 2016), but by late 2014, the vast majority of the Syrian refugees moved to the bigger cities (İçduygu, 2015). Today, Syrian refugees live across Turkey (Ministry of Interior, Directorate General of Migration Management, 2017), leaving only 13% of the Syrian refugees in refugee camps (Erdoğan, 2014).

In the beginning of their arrival to Turkey, Syrian refugees were welcomed and considered as “guests” (Kaya, 2016; Tolay, 2014). Turkey enacted a new Temporary Protection Legislation in 2014, which grants Syrian refugees a legal stay in Turkey until safe return is possible, and entry to social services like health, education, and labour market (İçduygu, 2015). Yet there are also political and public discussions about the challenges that refugees pose, and the possible naturalization of Syrian refugees fuels negative reactions among the Turkish public (Erdoğan, 2015). Reflecting these developments, there are people having a more welcoming attitude towards Syrian refugees and people having a more negative attitude (Erdoğan, 2014; Konda Research & Consultancy, 2016). For example, there are many Turkish citizens who view Syrian refugees as an economic and as a security threat. In a public opinion survey, 60% of the respondents indicated that “Cities are less secure because of the presence of refugees,” 58% of the respondents agreed with “Refugees are affecting Turkish economy negatively,” and 61% thought that “There are less employment opportunities because of the presence of refugees” (Konda Research & Consultancy, 2016). However, there are also more positive attitudes based on humanitarian principles. For example, a public opinion survey showed that 46.6% of Turkish respondents agreed with the statement that “Admission of Syrians without any discrimination regarding their language, religion, and ethnic background is a humanitarian obligation on our part.” Another public survey revealed that around 56% agreed that it is a humanitarian duty to accept Syrian refugees and that there should not be any discrimination towards them (Konda Research & Consultancy, 2016).

### 1.2 Positive and negative behavioural intentions

Most studies examine people's (prejudicial) beliefs and feelings towards refugees and immigrants (Esses, Dovidio, Jackson, & Armstrong, 2001; Esses et al., 2017; Plener, Groschwitz, Brähler, Sukale, & Fegert, 2017; Schweitzer, Perkoulidis, Krome, Ludlow, & Ryan, 2005) and do not consider behavioural intentions. Yet these intentions are closest to people's actual behaviour, and research has demonstrated, for example, that protest intentions and actual behaviour tend to be associated (Van Zomeren, Postmes, & Spears, 2008). Refugees receive various sorts of assistance, but often they also face discrimination and social exclusion. Thus, both positive and negative behavioural intentions are important to study, and people might demonstrate a mixture of both. The positive–negative asymmetry in intergroup relations indicates that positive evaluations and intentions differ from negative evaluations and intentions (Buhl, 1999; Mummendey & Otten, 1998; Otten & Mummendey, 2000). A less positive orientation towards an outgroup compared with the ingroup is consistently found on evaluation dimensions and behaviour with positive connotations, but not on negatively valued dimensions or negative behaviour. One reason for this is that, in general, the differential evaluation of negative traits and behaviour is socially less acceptable than the differential evaluation of positive traits and behaviour (see Mummendey & Otten, 1998; Otten & Mummendey, 2000). Wenzel and Mummendey (1996) showed that negative valence increases the social concern with the legitimacy and appropriateness of unequal group distinctions. Furthermore, the domains of positive and negative actions and behavioural intentions have been found to involve different moralities with distinct motivational and regulatory systems (Janoff‐Bulman, Sheikh, & Hepp, 2009). Positive behaviour that focuses on advancing other's well‐being raises questions of prescriptive morality that indicates what one should do, whereas negative behaviour involves proscriptive morality that indicates what one should *not* do. Prescriptive morality is abstract, commendatory, and discretionary, whereas proscriptive morality is concrete, condemnatory, and duty‐based resulting in greater moral blame (Janoff‐Bulman et al., 2009). This means that negative actions and behavioural intentions against disadvantaged people is likely to be morally more difficult than not assisting or helping them. In the present study and considering the normative and moral implications, we expected that Turkish respondents make a distinction between positive and negative behavioural intentions towards Syrian refugees.

### 1.3 National identification and the mediating role of perception of threat

There is a substantial empirical literature that shows that stronger national identification is associated with more negative attitudes towards immigrants and minority groups (e.g., Blank & Schmidt, 2003; De Figueiredo & Elkins, 2003; Esses, Dovidio, Semenya, & Jackson, 2004; Esses, Wagner, Wolf, Preiser, & Wilbur, 2006). Higher compared with lower national identifiers are more focused upon and concerned about their national ingroup. According to self‐categorization theory (Turner, Hogg, Oakes, Reicher, & Wetherell, 1987), group identity functions as a group lens that increases the sensitivity of people about anything that could harm their in‐group. Higher identifiers tend to view the social world in terms of their group membership and therefore are more focused upon and stronger inclined to perceive possible threats. For example, a research among native Dutch participants found that national identification was positively associated with perceived outgroup threat and via threat to a stronger rejection of cultural rights for immigrant minorities (Verkuyten, 2009). Further, in the context of Israel and Germany, it was found that perceived socio‐economic threat fully mediated the association between national identification and exclusionary attitudes towards immigrants (Hochman, Raijman, & Schmidt, 2016). Following self‐categorization theory and these empirical findings, we expected that higher national identifiers will perceive Syrian refugees as more threatening to Turkish security and identity and as a result will have more negative and less positive behavioural intentions towards these refugees.

### 1.4 Humanitarian concerns

People can not only feel threatened and demonstrate prejudicial reactions towards refugees, but they also can act favourably towards this group. There are many examples of assistance and help being provided to refugees and, as indicated by the Turkish opinion polls discussed, these acts can be based on humanitarian concerns. Humanitarian concerns involve a sense of compassive care and moral responsibility for the welfare of fellow human beings, especially when they are in need. These concerns have been found to be associated with stronger support for refugees (Verkuyten, Altabatabaei, & Nooitgedagt, 2018) and are based on a shared humanity. The human level of identity defines Turks and Syrian refugees as forming part of the same humanity. The common in‐group identity model (Gaertner & Dovidio, 2000) suggests that a superordinate identity makes subgroup boundaries less salient and that former outgroup members will be part of the in‐group resulting in more favourable attitudes and behaviours (e.g., Beaton & Deveau, 2005). There is extensive empirical evidence supporting this model in a range of settings and among various groups (see Gaertner & Dovidio, 2000), including the positive effect of shared humanity for attitudes towards asylum seekers (e.g., Nickerson & Louis, 2008). Humanitarian concern reflects an identification with other human beings with the related moral responsibility to help them in times of need. Shared humanity has been found to be associated with the endorsement of human rights, intergroup empathy, and providing humanitarian aid and relief (e.g., McFarland, Webb, & Brown, 2012; Reysen & Katzarka‐Miller, 2013). Thus, it can be expected that stronger humanitarian concern is associated with stronger positive behavioural intentions towards these refugees and weaker negative behavioural intentions.

In addition to humanitarian concern being expected to be associated with behavioural intentions towards refugees, we also examined whether these concerns moderate the association between feelings of outgroup threat and positive and negative behavioural intentions. In relation to the so‐called refugee crisis, host societies often struggle with finding a balance between humanitarian considerations and societal interests (Verkuyten et al., 2018). People might not only be concerned about the threats that refugees can pose to the unity and safety of society but can also feel a sense of compassion and moral responsibility towards refugees. This could mean that the expected link between perceived threats and behavioural intentions depends on the level of humanitarian concerns. The perception of threat might be less strongly associated with behavioural intentions among individuals with stronger humanitarian concerns. Research has demonstrated that moral norms can influence the expression or suppression of prejudices (Crandall, Eshleman, & O'Brien, 2002; Thijs, Gharaei, & de Vroome, 2016). When people feel threatened by an outgroup but also consider members of this outgroup as fellow human beings, this might increase the intention to act positively towards them and suppress the intention to act negatively. Humanitarian concerns make it possible that feelings of threat are less likely to translate in lower positive behavioural intentions and in higher negative behavioural intentions. Thus, the negative association between threat and positive behavioural intentions can be expected to be weaker for Turkish participants with stronger humanitarian concerns. Correspondingly, for these respondents, the positive association between threat and negative behavioural intentions can be expected to be weaker.

### 1.5 The current study

We examined whether the perception of threat mediates the association between national identification and negative and positive behavioural intentions of Turkish citizens towards Syrian refugees and whether the role of threat depends on the level of humanitarian concern. First, Turkish respondents were expected to differentiate between positive and negative behavioural intentions. Second, higher national identification was expected to be associated with more negative and less positive behavioural intentions towards Syrian refugees, and perception of threat was expected to mediate this relationship, because higher identifiers will perceive Syrian refugees more strongly as a threat to Turkish identity and security. Third, Turkish respondents with stronger humanitarian concerns were expected to have more positive behavioural intentions and less negative behavioural intentions towards Syrian refugees. Fourth, these concerns were expected to moderate the association between perception of threat and behavioural intentions towards Syrian refugees.

## 2 METHOD

### 2.1 Participants

This study was conducted with 605 Turkish citizens (43.6% male and 56.4% female) between 18 to 81 years of age (*M* = 39.6, *SD* = 14.4). The addresses of the participants were selected by the Turkish Statistical Institute from six cities in Turkey, which varies in terms of the ratio of Syrian refugees to the population of the city. The selected cities were Istanbul (33.4% of participants), Antalya (22.3%), Gaziantep (13.7%), Adana (13.4%), Samsun (8.9%), and Kilis (8.3%). Of the participants, 87.6% ethnically self‐identified as Turks, 6.9% as Kurds, 1.5% as Arabs, 0.8% as Zaza, and 3.1% was from other ethnic groups. The ratio of the Syrian refugee population to the population of the cities they reside differs with Samsun and Antalya having a relatively low number of Syrian refugees (0.1% and 0.5%, respectively), Adana and İstanbul having a somewhat higher ratio of Syrian refugees (2.5% and 2.6%, respectively), and Gaziantep and Kilis having a relatively high number of Syrian refugees (14% and 41%, respectively; Turkish Statistical Institute, 2016 and Ministry of Interior, Directorate General of Migration Management, 2017). The study was conducted by the research company Optimar in May and June 2015. The respondents participated in the survey voluntarily in their home, and it took about 20 to 25 min to complete the survey. A survey taker administered the paper‐and‐pencil questionnaires.

### 2.2 Measures

The dependent variable—*positive behavioural intentions*—was measured by asking the respondents to indicate the likelihood (5‐point scales, 1 = *very unlikely* and 5 = *very likely*) of engaging in seven positive behaviours: “Help a Syrian refugee when I am asked to,” “Share the same table with a Syrian refugee,” “Become friends with a Syrian refugee,” “Add a Syrian refugee on Facebook as friends,” “Participate in a protest in favor of Syrian refugees,” “Sign a petition in favor of Syrian refugees”, and “Donate money for improving the living conditions of Syrian refugees.”

The other dependent variable—*negative behavioural intentions*—was measured in terms of the likelihood of engaging in two forms of negative behaviour: “Participate in a protest against Syrian refugees,” and “Sign a petition *against* Syrian refugees.” All items were rated on 5‐point scales (1 = *very unlikely* and 5 = *very likely*).

We expected that the respondents make a distinction between positive and negative behavioural intentions. Factor analysis with maximum likelihood extraction and oblimin rotation showed that the positive behavioural intentions and negative behavioural intentions items loaded on two separate factors. Positive behavioural intentions items loaded high on the first factor (>.71; on the second factor highest load = .18) that explained 54.12% of the variance. The two negative behavioural intentions items loaded very high on the second factor (>.96; highest load on the first factor = −.02) that explained 19.70% of the variance. An average score for positive behavioural intentions was computed (α = .93) and also an average score for the two negative behavioural intentions (*r* = .95, *p* < .001) with a higher score indicating more negative behavioural intentions. As shown in Table 1, both measures were negatively but not strongly (*r* = −.22, *p* < .001) associated, which further supports their empirical distinctiveness.

**Table 1:** Correlations, means, and standard deviations of the main constructs

Constructs	1	2	3	4	*M*	*SD*
1. National identification	—				4.29	.76
2. Perception of threat	.19**	—			3.65	1.04
3. Humanitarian concerns	−.04	−.38**	—		3.10	1.08
4. Positive behavioural intentions	−.17**	−.59**	.57**	—	2.21	1.07
5. Negative behavioural intentions	.06	.32**	−.37**	−.22**	2.05	1.41

** *p* < .01.

*Turkish national identification* was measured with three items that did not focus on ethnicity but rather on Turkish citizenship (*Turkiyeli*), which includes various ethnic groups. These items have been used in previous studies in Turkey (e.g., Çelebi, Verkuyten, Köse, & Maliepaard, 2014): “I am proud to be a citizen of Turkey,” “Being a citizen of Turkey is an important part of who I am,” “I strongly feel that I am a citizen of Turkey.” All items were rated on a 5‐point scales (1 = *certainly not agree* and 5 = *certainly agree*), and an average score of these items was computed (α = .92).

*Perceived outgroup threat* was assessed by focusing on symbolic and security threat using items that were adapted from previous studies (Stephan, Diaz‐Loving, & Duran, 2000; Stephan & Stephan, 1996): “The cultural identity of Turkey is being threatened by the increasing number of Syrian refugees,” “The norms and values of Turkey are being threatened due to the presence of Syrian refugees,” “The Syrian refugees are undermining the culture of Turkey,” “I worry that violent conflicts between Syrian refugees and people living in Turkey may happen,” “I worry about the rise of stealing, begging, and attacking of the people living in Turkey,” and “I worry about Syrian refugees spreading diseases.” All items were rated on a 5‐point scales (1 = *certainly not agree* and 5 = *certainly agree*). An average score of these items was computed (α = .91).

*Humanitarian concerns* was measured in relation to Syrian refugees and with three items that focused on compassive care and felt moral responsibility: “I pity Syrian refugees because they are also humans,” “I should help Syrian refugees because they are also humans,” and “As a human being I feel responsible for taking care of Syrian refugees.” All items were rated on 5‐point scales (1 = *certainly not agree* and 5 = *certainly agree*), and an average score was computed (α = .85).

## 3 RESULTS

### 3.1 Descriptive findings

As shown in Table 1, the mean scores of positive and negative behavioural intentions were similar, *t*(590) = 1.91, *p* = .056, and both were significantly below the neutral midpoint of the scale *t*(592) = −18.12, *p* < .001 and *t*(601) = −16.51, *p* < .001, respectively. This indicates that Turkish respondents reported no clear inclination for positive behaviour but also not for negative behaviour towards Syrian refugees. One sample *t* test showed that the mean scores of national identification, humanitarian concerns, and perception of threat were significantly above the midpoint of the scales *t*(597) = 41.66, *p* < .001; *t*(603) = 2.29, *p* = .022; and *t*(594) = 15.10, *p* < .001, respectively.

National identification and perception of threat were negatively correlated with positive behavioural intentions. Humanitarian concern was positively correlated with positive behavioural intentions. As shown in Table 1, higher national identification and stronger perception of threat were associated with more negative behavioural intentions, whereas humanitarian concern was negatively associated with negative behavioural intentions.

### 3.2 Positive behavioural intentions

To test the moderated mediation hypothesis presented in Figure 1, we used Model 14 in process macro (Preacher, Rucker, & Hayes, 2007) with 10,000 bootstraps. However, we first conducted a regression analysis to examine whether the demographic variables age, gender, city, and ethnicity (Turkish versus non‐Turkish) should be included as control variables.^1^ This analysis indicated that gender, city, and ethnicity were significant predictors, and therefore, these demographics were added as control variables in the moderated mediation analysis.

**Figure 2:**
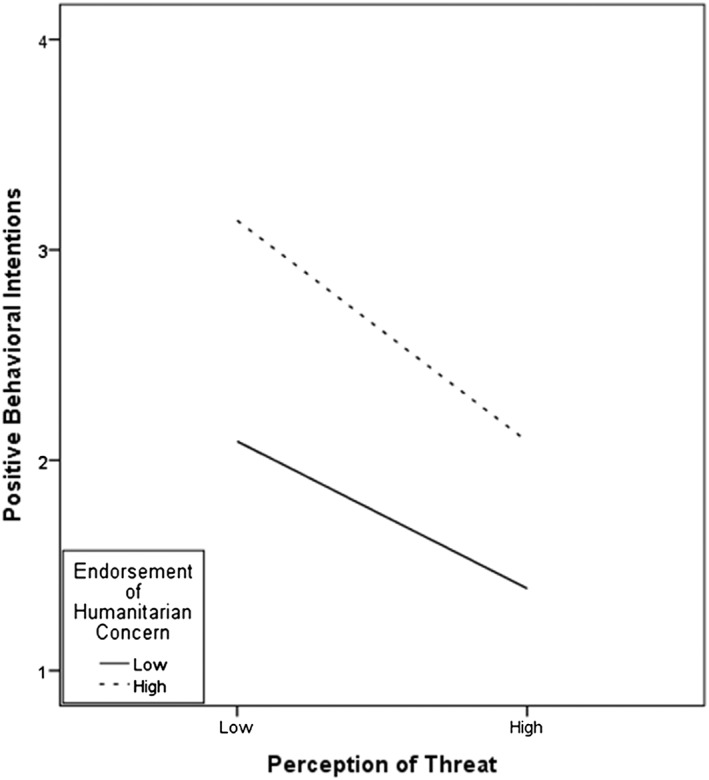
Interaction effect between perception of threat and endorsement of humanitarian concern on positive behavioural intentions

Results of this analysis showed that higher national identification was associated with stronger perception of threat, *B* = .21, *SE* = .06, *t* = 3.48, *p* < .001, 95% CI [.091, .327], and as shown in Table 2, higher threat was associated with lower positive behaviour intentions, *B* = −.41, *SE* = .03, *t* = −11.94, *p* < .001, 95% CI [−.474, −.340], whereas stronger humanitarian concern was associated with more positive behaviour intentions, *B* = .40, *SE* = .03, *t* = 12.64, *p* < .001, 95% CI [.338, .463]. The direct effect of national identification on positive behavioural intentions was not significant *B* = −.07, *SE* = .05, *t* = −1.51, *p* = .132 with a 95% CI [−.157, .021], but the indirect effect of national identification through perception of threat on positive behavioural intentions was −.015, which is significant as the 95% CI [−.034, −.003] does not contain zero. This mediation effect was qualified by a significant interaction between perception of threat and humanitarian concern, *B* = −.07, *SE* = .03, *t* = −2.47, *p* = .014 with a 95% CI [−.126, −.014]. Unexpectedly, however, and as shown in Figure 2, simple slope analyses of the interaction effect indicated that the conditional indirect effect of national identification on positive behavioural intentions through perception of threat was somewhat stronger for relatively high level of humanitarian concern (+1 *SD*) *B* = −.10, *SE* = .03, *p* = .007, 95% CI [−.171, −.038], compared with low level of humanitarian concern (−1 *SD*) *B* = −.07, *SE* = .03, *p* = .007, 95% CI [−.127, −.027]. Thus, although the difference in association is small, higher threat tended to have a stronger association with less positive behavioural intentions for those participants who had stronger humanitarian concerns compared with those with weaker humanitarian concerns.

**Table 2:** Multiple Regression Analyses Predicting Positive and Negative Behavioral Intentions

Variables	Positive Behavioral Intentions	Negative Behavioral Intentions
	β *(SE)*	
Gender	.14(.07)*	‐.02(.11)
City	‐.08(.02)***	‐.03(.04)
Non‐Turkish	‐.14(.10)	‐.68(.16)***
National identification	‐.07(.05)	.08(.07)
Humanitarian concern	.40(.03)***	‐.33(.05)***
Perception of threat	‐.41(.03)***	.34(.06)***
Perception of threat x humanitarian concern	‐.07(.03)*	‐.15(.05)**
*R* ^*2*^	.51	.20

* *p* < .05.

** *p* < .01.

*** *p* < .001.

### 3.3 Negative behavioural intentions

**Figure 3:**
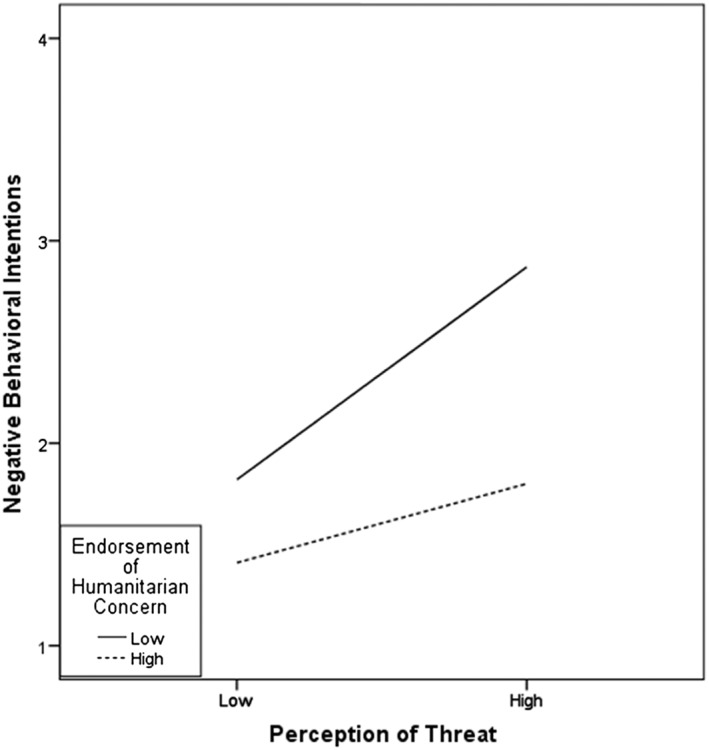
Interaction effect between perception of threat and endorsement of humanitarian concern on negative behavioural intentions

In a regression analysis, gender, city, and ethnicity were significant predictors of negative behavioural intentions, and the different variables explained a lower amount of the total variance than for positive behavioural intentions. We added the demographic variables as controls in the moderated mediation analysis. Findings of this analysis indicated that higher national identification was associated with stronger perception of threats, *B* = .23, *SE* = .06, *t* = 3.97, *p* < .001, 95% CI [.116, .343], and as shown in Table 2, higher perception of threat was associated with more negative behavioural intentions, *B* = .34, *SE* = .06, *t* = 5.99, *p* < .001, 95% CI [.227, .449]. Stronger humanitarian concern was associated with lower negative behavioural intentions, *B* = −.33, *SE* = .05, *t* = −6.38, *p* < .001, 95% CI [−.434, −.230]. Furthermore, the perception of threat was found to mediate the relation between national identification and negative behavioural intentions. The direct effect of national identification on negative behavioural intentions was not significant *B* = −.08, *SE* = .07, *t* = 1.11, *p* = .266, whereas the indirect effect of national identification through perception of threat was −.034, which was significant as the 95% CI [−.069, −.011] does not contain zero. This mediation was again qualified by a significant interaction effect between perception of threat and humanitarian concern, *B* = −.15, *SE* = .05, *t* = −3.10, *p* = .002 with a 95% CI [−.238, −.054]. As expected and as shown in Figure 3, the conditional indirect effect of national identification on negative behavioural intentions through perception of threat was less strong for high humanitarian concern (+1 *SD*) *B* = .04, *SE* = .02, *p* = .001, 95% CI [.016, .088], compared with low humanitarian concern (−1 *SD*) *B* = .11, *SE* = .04, *p* = .001, 95% CI [.050, .201].

### 3.4 Additional analyses

Although we had theoretical reasons for testing our moderated mediation model, we conducted two additional analysis to investigate alternative models. Table 1 shows that national identification was not associated with humanitarian concerns, which means that the latter does not mediate the effect of the former on behavioural intentions. However, humanitarian concerns might moderate the relationship between national identification and perceived threat. The test of this model (Model 7, Preacher et al., 2007) showed that the interaction between national identification and humanitarian concern on the perception of threat was not significant (*B* = −.01, *SE* = .05, *p* = .818 for positive behavioural intentions and *B* = −.02, *SE* = .05, *p* = .715 for negative behavioural intentions).

Additionally, we used Model 2 (Preacher et al., 2007) to examine whether humanitarian concern and perceived threat both moderate the direct link between national identification and positive and negative behavioural intentions. Results showed that perception of threat moderated the association between national identification and positive behavioural intentions (*B* = .10, *SE* = .04, *p* = .02), whereas humanitarian concern did not moderate the association between national identification and positive behavioural intentions (*B* = −.01, *SE* = .04, *p* = .760). Furthermore, there were no significant interaction effects with national identification for negative behavioural intentions (with perception of threat, *B* = .10, *SE* = .07, *p* = .123; with humanitarian concern, *B* = −.03, *SE* = .06 *p* = .689).

## 4 DISCUSSION

The so‐called “refugee crisis” has led to fierce societal debates, and many people have ambivalent feelings about the refugee question. On the one hand, people tend to sympathize and empathize with the difficult plight of refugees, and there are many voluntary and organized initiatives to offer help and support. On the other hand, the arrival of refugees makes people insecure and feel threatened leading to opposition. Turkey is at the forefront of the “refugee crisis” hosting almost 3 million Syrian refugees, and our research is one of the first to assess Turkish citizens' positive and negative behavioural intentions towards these refugees.

We found that participants make a distinction between positive and negative behavioural intentions and that higher national identification was associated with more negative behavioural intentions and less positive behavioural intentions. In addition, these associations were explained by perceived threat. These findings are in line with previous studies (Hochman et al., 2016; Verkuyten, 2009) and with self‐categorization theory (Turner et al., 1987). This theory argues that group identity—in this case, national identification—functions as a group lens that increases the sensitivity of people about anything that could harm their ingroup. This pattern of findings suggests that Turkish citizens who have higher national identification perceive Syrian refugees as more threatening to the national identity and security and as a result have more negative and less positive behavioural intentions towards Syrian refugees.

In contrast to research on the importance of national identification and threat for outgroup attitudes, few studies have examined the role of humanitarian concerns (Nickerson & Louis, 2008; Verkuyten et al., 2018). The findings of the current study show that stronger humanitarian concern was associated with less negative behavioural intentions and more positive intentions towards Syrian refugees. Humanitarian concern implies identification with other human beings with the related moral responsibility, which is likely to translate in more positive behavioural intentions and less negative ones.

In addition to the direct effect of humanitarian concerns, we examined the possibility that these concerns make perceived threat less important for one's behavioural intentions towards Syrian refugees. For negative behaviours, the results indeed show that humanitarian concern has a small buffering effect on the association between threat and behavioural intention. This indicates that when people feel threatened by refugees but at the same time also have a sense of compassive care and moral responsibility for the welfare of Syrians as fellow human beings, this reduces their intention to act negatively towards this outgroup. Thus, Syrian refugees can be perceived as a threat but when people also feel a humanitarian concern, they are less inclined to protest and rally against them.

Suprisingly, however, we also found that higher perceived threat is more strongly associated with lower positive behavioural intentions when humanitarian concern was relatively strong. Thus, respondents who felt more threatened and also more strongly felt pity and a moral responsibility towards Syrians as fellow human beings indicated a lower inclination to help and assist these refugees. This is an intriguing finding that might indicate that under conditions of perceived threat, an appeal to humanitarian concerns can backfire. Humanitarian considerations have been found to be especially important for the support of refugees among those who do not find the topic of immigration very important (Verkuyten et al., 2018). People who feel threatened by refugees can be expected to be concerned about immigration, and for them, a humanitarian appeal might lead to reactance. Yet, another, perhaps more likely, interpretation is to understand the interaction effect in the reversed way. What our findings then show is that feelings of threat can reduce the positive behavioural intentions that humanitarian concern implies. However, such a reversed interpretation cannot explain the interaction found for negative behavioural intentions. Thus, the pattern of findings for positive and negative behavioural intentions suggests that the interaction between perceived outgroup threat and humanitarian concern can work out differently and future studies should examine the different moralities involved in positive and negative behaviour in relation to refugees more closely (Janoff‐Bulman et al., 2009). Helping behaviour raises questions of prescriptive morality, whereas proscriptive rules underlie the blameworthiness of harmful or unfair behaviour. Although harming someone is almost always blameworthy, not helping others is not. Furthermore, it might be easier to reduce negative behavioural intentions because people view negative behavior as socially less acceptable, especially when humanitarian concerns are involved (Mummendey & Otten, 1998; Otten & Mummendey, 2000). In contrast, stimulating positive behaviour might be more difficult because it implies personal costs, such as time, effort, and investment.

### 4.1 Limitations

There are some limitations to the current study that we want to draw attention to. First, the data were collected from a variety of cities through a two‐stage clustering sampling method, which means that the findings cannot be generalized to the whole population of Turkey. Yet we managed to collect data across the country and in a society that, as a neighbouring country, hosts many Syrian refugees. Furthermore, we examined theoretically derived associations between key social psychological constructs. Future studies should try to collect more representative data and could use a longitudinal or experimental design (see Verkuyten et al., 2018) for systematically testing the proposed directions of influence.

Second, we measured behavioural intentions, so we do not know whether these translate into actual behaviour. Additionally, the fact that positive behavioural intentions were measured with seven items and negative behavioural intentions with two items and that the kind of behaviours differ means that we cannot compare the mean levels of intention. However, for a comprehensive understanding, it is important to consider both positive and negative behavioural intentions. Furthermore, pattern of associations can be examined, and these were central in our reasoning and statistical tests.

Third, we measured humanitarian concern specifically with regard to Syrian refugees and not in general terms. This could mean that there is some overlap with the questions on the intentions to help these refugees. However, we found associations with both positive and negative behavioural intentions. Further, in additional analyses without the humanitarian concern item that mentioned helping (“I should help Syrian refugees because they are humans”) yielded exactly the same results.^2^

Fourth, there are other possible processes and moderating conditions that might have an impact on the behavioural intentions towards Syrian refugees and that we did not examine. For example, level of income, educational level, political ideology, and intergroup contact might be important factors and conditions to consider. Furthermore, individual differences in, for example, social dominance orientation, right wing authoritarianism, and perceived competition for scarce resources are likely to be important (Esses et al., 2017).

## 5 CONCLUSION

In contrast to the existing research on (negative) beliefs and feelings towards refugees (Esses et al., 2017), we focused on both positive and negative behavioural intentions. Furthermore, we examined these intentions in relation to national identification and perceptions of threat as well as humanitarian concerns. We conducted our study in an underresearched national context that is highly relevant for understanding how people react towards the arrival of refugees. It was found that stronger national identification was associated with more negative behavioural intentions and less positive behavioural intentions towards Syrian refugees via perceived threat. Additionally, stronger humanitarian concern was associated with a stronger intention to help and support refugees and a weaker intention to protest against them. Furthermore, the findings suggest that the combination of perceived threat and humanitarian concern can work out differently for positive and negative behavioural intentions towards Syrian refugees. This could mean that when people feel threatened by refugees, an emphasis on humanitarian concerns might not always have beneficial consequences for refugees: It might reduce negative behavioural intentions but also the inclination to offer help and support. This possibility has practical implications. Public campaigns and social policies that appeal to humanitarian concerns for improving intergroup relations between Syrian refugees and Turkish people should be managed cautiously. People might understand these campaigns and appeals as ignoring their genuine feelings of threat and as implying a moral accusation of failing to meet humanitarian standards. To them, these campaigns and appeals might be threatening to their sense of moral self, which leads to justifications of their behaviour (Ellemers, 2017). This means that people's feelings of threat should be taken seriously and not dismissed as being misguided and prejudicial. An appeal to humanitarian concerns might be most effective when feelings of threat are considered and reduced. This means that future studies should investigate the correlates and causes of feelings of threat and the ways in which these hamper the inclination to offer help and support. In doing so, it is important to develop a more detailed understanding of the similar as well as different processes involved in positive and negative behavioural intentions.

## CONFLICT OF INTEREST

Author Şenay Yitmen declares that she has no conflict of interest. Author Maykel Verkuyten declares that he has no conflict of interest.

## ENDNOTES

1 Additional analyses without the control variables did not change the findings of the moderated mediation model for both the positive and negative behavioural intentions. For example, the direct effect of national identification on positive behavioural intentions was significant *B* = −.11, *SE* = .04, *t* = −2.52, *p* = .012, whereas the indirect effect of national identification through perception of threat was −.018, which is significant as the 95% CI [−.037, −.005] does not contain zero. This mediation was again qualified by a significant interaction effect between perception of threat and humanitarian concerns, *B* = −.08, *SE* = .03, *t* = −2.67, *p* = .008 with a 95% CI [−.133, −.020].

2 These findings are available upon request.
